# The contribution of the immune system to parturition

**DOI:** 10.1155/S0962935196000233

**Published:** 1996-06

**Authors:** R. De Jongh, Ph. Jorens, I. Student, R. Heylen

**Affiliations:** 1 Department of Anaesthetics Intensive Care Unit Ziekenhuis Oost-Limburg Campus St.-Jan, Schiepse Bos 6 Genk B 3600 Belgium; 2 Department of Intensive Care Medicine University Hospital Antwerp Belgium; 3 Zentrum für Anästhesiologie Abteilung IV Medizinischen Hocheschule Hannover Germany

## Abstract

The immune system plays a central role before and during parturition, including the main physiological processes of parturition: uterine contractions and cervical ripening. The immune system comprises white blood cells and their secretions. Polymorphonuclear cells and macrophages invade the cervical tissue and release compounds, such as oxygen radicals and enzymes, which break down the cervical matrix to allow softening and dilatation. During this inflammatory process, white blood cells undergo chemotaxis, adherence to endothelial cells, diapedesis, migration and activation. Factors that regulate white blood cell invasion and secretion include cytokines such as tumour necrosis factor and interleukins. Glucocorticoids, sex hormones and prostaglandins, affect cytokine synthesis. They also modulate the target cells, resulting in altered responses to cytokines. On the other hand, the immune system has profound effects on the hormonal system and prostaglandin synthesis. In animals, nitric oxide has marked effects on uterine quiescence during gestation. At the same time, it plays an important role in regulating the vascular tone of uterine arteries and has anti-adhesive effects on leukocytes. Cytokines are found in amniotic fluid, and in maternal and foetal serum at term and preterm. Several intrauterine cells have been shown to produce these cytoldnes. Since neither white blood cells, cytokines nor nitric oxide seem to be the ultimate intermediate for human parturition, the immune system is an additional but obligatory and underestimated component in the physiology of delivery. Scientists, obstetricians and anaesthesiologists must thus be aware of these processes.


Mediators of Inflammation 5, 173–182 (1996)

### The contribution of the immune system to parturition

R. De Jongh,[1,CA] Ph. Jorens,[2] I. Student[3] and R. Heylen[1]

[1]Department of Anaesthetics, Intensive Care Unit, Ziekenhuis Oost-Limburg, Campus St.-Jan, Schiepse Bos 6, B 3600 Genk, Belgium; [2]Department of Intensive Care Medicine, University Hospital Antwerp, Belgium; [3]Zentrum für Anästhesiologie, Abteilung IV, Medizinischen Hocheschule Hannover, Germany

[CA]Corresponding Author

**The immune system plays a central role before and during parturition, including the main physiological processes of parturition: uterine contractions and cervical ripening. The immune system comprises white blood cells and their secretions. Polymorphonuclear cells and macrophages invade the cervical tissue and release compounds, such as oxygen radicals and enzymes, which break down the cervical matrix to allow softening and dilatation. During this inflammatory process, white blood cells undergo chemotaxis, adherence to endothelial cells, diapedesis, migration and activation. Factors that regulate white blood cell invasion and secretion include cytokines such as tumour necrosis factor and interleukins. Glucocorticoids, sex hormones and prostaglandins affect cytokine synthesis. They also modulate the target cells, resulting in altered responses to cytokines. On the other hand, the immune system has profound effects on the hormonal system and prostaglandin synthesis. In animals, nitric oxide has marked effects on uterine quiescence during gestation. At the same time, it plays an important role in regulating the vascular tone of uterine arteries and has anti-adhesive effects on leukocytes. Cytokines are found in amniotic fluid, and in maternal and foetal serum at term and preterm. Several intrauterine cells have been shown to produce these cytokines. Since neither white blood cells, cytokines nor nitric oxide seem to be the ultimate intermediate for human parturition, the immune system is an additional but obligatory and underestimated component in the physiology of delivery. Scientists, obstetricians and anaesthesiologists must thus be aware of these processes.**

**Key words**: Inflammation, Interleukin, Labour, Nitric oxide, Parturition

### Introduction

The effect of anaesthesia, stress and pathological disorders on the process of labour and the mode of delivery remains controversial.[1] Because randomization and clinical research in this field is limited by ethical considerations, the physiology of parturition, especially the underlying autocrine, paracrine and endocrine control must be understood. Therefore, physicians should be familiar with the physiological processes which contribute to myometrial contractions and cervical ripening, and must be aware of recent trends and research.

Until labour, cervical dilatation and particularly labour are suppressed. During parturition, forceful myometrial contractions propel the foetus through the dilated and ripened cervix and vaginal tract. Several cascade pathways including the hormonal system, prostaglandins and the immune system, contribute to elicit or augment cervical ripening and labour.

Hormones such as progesterone, oestrogens and oxytocin may inhibit or induce physiological processes in the uterus, but they fail to explain the entire mechanism of parturition. The discovery of prostaglandins and their contribution to labour was an obstetric revolution with dramatic clinical impact. Prostaglandins are obligatory in parturition, but they are not considered the sole intermediate modulator of this process.

Cervical ripening is an inflammatory process,[2] since polymorphonuclear cells (PMNs) and macrophages are found in abundance in cervical tissue. Cytokines, which are small intercellular signal peptides of the immune system, are found

© 1996 Rapid Science Publishers in varying concentrations in amniotic fluid, foetal and maternal serum before and during parturition.[3–8] They are capable of modulating prostaglandin production.[9–12] Several foetal and maternal cells produce these cytokines.[13–16] The fact that inflammation contributes to delivery is suggested by the finding of irreversible labour and cervical ripening with an increased production of cytokines during intra-uterine infection.[9–11,17] Moreover, intrauterine but not intraperitoneal bacterial inoculum resulted in preterm delivery within 2 days.[18]

The aim of this article is to propose some immunological processes which might play a role during cervical ripening and labour. Initially we describe the function of the inflammatory cells, restricted to monocytes and polymorphonuclear cells (PMNs), and their secretion products including oxygen radicals, proteases and cytokines. Since the inflammatory cells have to migrate into tissues, a part of this article summarizes the leucocyte–endothelial cell interaction. Then the physiological result of inflammation of the cervix and the myometrium is described. In the last part of this article a summary is given of the complex interaction of inflammatory and neurohumoral pathways. Since research on parturients in this domain is sparse, most of the information dealing with PMNs, macrophages, cytokines and oxygen radicals originates from *in vitro* studies or from polytraumatized or septic patients or animals. Furthermore, the article has not the intention to be complete, but describes basic physiological pathways leading to an understanding of more specialized articles.

### The immune system

The immune system is classically divided into an innate branch and an acquired branch. The latter is mainly composed of lymphocytes and immunoglobulins, and the former mainly of monocytes, macrophages, PMNs and natural killer cells (NK). The protein components of the innate branch include complement factors and acute phase proteins (APP).[19] The cells of the innate branch act by producing cytokines, proteases and oxygen radicals.

*Monocytes and macrophages:* Monocytes and macrophages are part of the mononuclear phagocyte system.[20] The macrophage can interact with its environment through the binding of molecules to specific functional membrane surface receptors, e.g. for cytokines or complement fragments. The potent antimicrobial, tumoricidal and tissue destructing activity of stimulated macrophages is explained by the local release of several molecules, including (oxygen) radicals; proteolytic enzymes such as lysozyme, collagenase and elastase; and cytokines such as tumour necrosis factor (TNF) and interleukin-1 (IL-1). Macrophages can release chemotactic factors for other inflammatory cells. The most important factor is interleukin-8 (IL-8), but leukotrienes and platelet activating factor (PAF) also have chemotactic properties.[21] On the other hand, macrophages can release factors that inhibit PMN chemotaxis,[22,23] resulting in the accumulation of PMNs around macrophages.

During inflammation, the localization of macrophages in the tissues allows these cells to communicate with other adjacent inflammatory cells, endothelial cells and fibroblasts. Of particular interest is the 'decidua-macrophage connection', because the decidua is enriched with bone-marrow-derived macrophages.[24,25] Moreover, decidual cells have macrophage-like functional characteristics, e.g. the production of PAF and several cytokines after endotoxin stimulation.[26]

*Polymorphonuclear cells:* PMNs possess three major types of granules containing over 20 different enzymes. After stimulation, e.g. by cytokines or complement factors, PMNs degranulate with a release of proteases and formation of oxygen radicals, resulting in tissue destruction. The extent of degranulation or respiratory burst by different stimulators has not been clarified fully.[27] Moreover, after stimulation, PMNs can synthesize many cytokines.

Because of the presence of large amounts of PMNs and macrophages in the near-term cervix,[2,28] and their capacity to produce proteases, cytokines and oxygen radicals, these cells must form a requisite step in the inflammatory process of ripening the cervix.

*Proteases:* Proteases are discussed under the heading 'cervical ripening'.

*Oxygen radicals:* Oxygen radical formation occurs during normal cell function, but in normal conditions they are neutralized or cleared. Sources of oxygen radicals which can result in tissue damage, with respect to parturition, include:

- Various metabolic steps of eicosanoid metabolism. Toxic oxygen radicals can, by themselves, stimulate eicosanoid metabolic pathways.[29,30]
- NADPH dehydrogenase and ubiquinone–cytochrome b complex of the mitochondrial electron transport chain. This process is upregulated in pathological conditions.[31]
- Ischaemia and particularly reperfusion, concomitant with the conversion of the xanthine substrates to uric acid.[32] Short-lasting but recurrent uterine ischaemia, followed by reperfusion, occurs during every myometrial contraction.

- Activation of the PMN (and macrophage) plasma membrane NADPH oxido-reductase enzyme complex, in response to endotoxins and cytokines.[33,34]

Oxygen is converted to its toxic metabolites: viz. superoxide anion ($O_2^-$), hydrogen peroxide ($H_2O_2$) and hypochlorous acid. The major targets of oxygen radicals, with subsequent destruction, include: lipids[35] (cellysis, cytotoxic and mutagenic metabolites), nucleic acids[36] (mutagenesis and carcinogenesis) and particularly proteins[37] (destruction, enzymatic dysfunction). Moreover, adhesion molecules on leukocytes are upregulated by some oxygen radicals. Several antioxidants are known; however, to date none of these agents have been used extensively in clinical practice. Probably the higher frequency of cervix carcinoma present in multiparous compared to nulliparous women is explained by the oxygen radicals formation during cervical ripening.

*Cytokines:* Cytokines are low molecular weight proteins, secreted in very small amounts by a variety of cell types. They modify the behaviour of other cells and are active in very low concentrations. One cytokine may have different effects, depending on the type of target cell, the concentration and the interaction with other cytokines. All cytokines have their specific membrane receptor(s), which can be shed and sometimes measured in the plasma. Until now it can only be assumed that cytokine receptors (and thus the cytokine effect) found intrauterine during delivery are changed in number, whether by cellular upregulation whether by binding of the receptor from the shedded plasma pool.

*In vitro* TNF, IL-6 and IL-8 are produced by several intrauterine cells after stimulation with endotoxin or other cytokines. Intrauterine sources of cytokines include decidua,[14,26,38] inflammatory cells, placental villi,[39] chorion[15] and endothelial cells. Normal spontaneous labour at term is associated with a moderate rise in the amount of TNF, IL-1, and especially IL-6 in amniotic fluid.[3–6] This rise is more pronounced during intrauterine infection, where a correlation was found between IL-6 and the cytokines TNF and IL-1.[8] This underscores the dependency of these cytokines or the same initiating pathways. In contrast to IL-6, neither TNF nor IL-1 is found in serum during labour. Some important cytokines and their possible role during parturition are discussed here.

*Interleukin-1.* Interleukin-1[40] exists in two forms, IL-1α and IL-1β, with only 26% homology. Almost all nucleated cells, but particularly macrophages and monocytes, are capable of producing IL-1. Stimuli for IL-1 production are: other cytokines such as IL-1 itself, TNF and interferons; endotoxins; antigen–antibody complexes; and the Fc regions of IgG and C5a. Inhibitors of IL-1 production include steroids, prostaglandin $E_2$ ($PGE_2$) and α-melanocyte stimulating hormone. An engodenously produced IL-1 receptor antagonist inhibits the effects of IL-1.

*In vitro*, the effects of IL-1 in the cytokine network are the amelioration of the synthesis of TNF, IL-1, IL-2, IL-6, PAF, prostacyclin, PGE2 and colony stimulating factor. IL-1 promotes the adhesiveness of neutrophils, monocytes and other white blood cells to the endothelial cell by upregulating adhesion molecules on leukocytes, and their ligands on endothelial cells.

*In vivo* IL-1 is pyrogenic and it plays a key role in the interaction of the immune and neuroendocrine systems, with increases of ACTH and corticosterone production. It causes mobilization of PMNs from the bone marrow, resulting in a neutrophilia. Hyaluronic acid and other glycosaminoglycans can stimulate IL-1 production, which in its turn can increase the collagenase and decrease the metalloproteinase inhibitor levels.[41] Considering the stimulatory effects of IL-1 on leukocytes, cytokine, and prostaglandin production, IL-1 forms a major step in the gestational inflammatory network. This was underscored by a study in which almost 100% of preterm mice aborted after systemic[42] or intra-amniotic[43] IL-1 administration. Interleukin-1 receptor antagonist prevented this IL-1 induced preterm parturition.[44]

*Interleukin-6.* Interleukin-6[45] can be produced by almost all nucleated cells. For macrophages, the most potent stimulator of IL-6 production is endotoxin, and for fibroblasts, IL-1 and TNF. Steroids and oestrogens are strong inhibitors of IL-6 production in both types of cells. The receptor of IL-6 is upregulated or downregulated by steroids, IL-1 and IL-6, depending on the type of cell studied.

IL-6, a pleiotropic cytokine, stimulates prostaglandin synthesis and it elicits, with the participation of steroids, the production of acute phase proteins by hepatocytes. These liver proteins have antiprotease, opsonizing and oxygen radical scavenging properties. It was therefore assumed that IL-6, in initiating this acute phase response, was responsible for limiting the inflammatory reaction to the injured area, viz. the cervix and the myometrium during parturition.

IL-6 was found in maternal and foetal serum and the levels correlated strongly with the duration of labour, thus probably also with the extent of cervical ripening and tissue destruction.[7] Since the induction of IL-6 synthesis via mRNA takes several hours, the serum IL-6 peak found immediately after vaginal delivery implies that inflammation during vaginal delivery is maximal before birth. Interestingly, considering that IL-6 induces fever, it was shown that patients with epidural analgesia have higher body temperatures after vaginal delivery than those without epidural analgesia.[46] However, in these studies the duration of labour was not included in the statistics as a dependent variable, although it is known that patients with prolonged labour are more likely to request epidural analgesia than the less affected 'controls'. IL-6 probably has a role in these temperature differences. Another study has demonstrated a relation between foetal IL-6 and maternal serum IL-6 levels, suggesting a maternally induced modification of foetal immune function and stress response.[47] IL-6 measured in plasma seems to be a biochemical marker of human preterm labour.[48]

*Tumour necrosis factor*. Tumour necrosis factor[49] is mainly produced by macrophages after stimulation by endotoxin and some cytokines. It promotes $PGE_2$ and collagenase production by fibroblasts, induces production of other cytokines such as IL-1, IL-6 and IL-8, is directly cytotoxic and is a direct stimulus for PMN respiratory burst.

*Interleukin-8*. Interleukin-8 is a strong chemotactic agent for PMNs but not for blood monocytes, a weak inducer of the respiratory burst[50] and a poor stimulator of elastase secretion by PMNs.[51] This suggests that chemotactic migration and the release of proteases by PMNs may be unrelated phenomena. IL-8 is produced by several cell types including macrophages, fibroblasts, chorion cells, decidual cells,[15] amnion cells,[16] and hepatocytes, when appropriately stimulated by members of the cytokine network, such as IL-1, TNF and endotoxin. In contrast to IL-8, neither TNF nor IL-1 seem to be directly chemotactic.

In contrast to the normal and stable plasma IL-8 levels during pregnancy and labour, IL-8 levels in amniotic fluid increase during gestation.[3] Infection and labour pain may trigger the production of IL-8 both at term and at preterm delivery.[4,5] In preterm labour, the IL-8 level in amniotic fluid is a more accurate predictor of histologic chorioamnionitis, tocolytic efficacy and early delivery than other microbiological tests (bacterial culture).[6] Since steroid hormones such as progesterone and corticosteroids are capable of influencing IL-8 production, and prostaglandins downregulate the threshold of PMN activation after IL-8 stimulation,[52] IL-8 might form the final common step of prostaglandin and steroid hormone action in parturition.[13,14]

Patients with chorioamnionitis have increased amniotic fluid levels of IL-1, IL-6, IL-8 and TNF. Moreover, this condition is known to be resistant to current available tocolytic therapy, indicating a dramatic abortive role of these cytokines.

*Leucocyte–endothelial cell interaction:* Margination of leukocytes is attributed to red blood cells which gather behind leukocytes in capillaries and push them toward the capillary wall once the vessel diameter exceeds 150% of that of the white blood cell. Then, due to low-affinity adhesive interaction between leukocytes and vascular endothelium on the one hand, and to the force of blood flow on the other hand, leukocytes start to roll. In inflamed tissue, leukocyte rolling frequently leads to a stationary state in which the leukocyte remains firmly attached to the endothelial surface for more than 30 s. These leukocytes can then leave the postcapillary venule by extending pseudopodia between apposing endothelial cells, entering the subendothelial space and the adjacent interstitial compartment.[53]

The basis for a selective appearance of different white cell types in association with different inflammatory reactions (cervicitis) is thought to be due to the production and release of unique chemoattractants at the site of inflammation, as well as to the presence and the number of specific receptors for these individual chemoattractants on selected cell types.

Leukocyte–endothelial cell adhesion is mediated by glycoproteins that belong to the selectin family of cell adhesion receptors. L-selectin is constitutively present on leukocytes, E-selectin is upregulated on the endothelial surface of postcapillary venules by cytokines and P-selectin is present on activated endothelium and platelets.[53] Each type of selectin possesses its own receptor, however all these ligands are not yet clearly defined. Selectins participate in rolling, whereas firm adhesion is possible by the interaction of integrins and their ligands.[54]

Circulating neutrophils are in the resting state if there is no systemic inflammation, but they contain β-integrin molecules constitutively on their surfaces. After PMN stimulation, these receptors are rapidly induced into a high state of avidity that promotes firm adhesion. At the same time, adhesomes, which contain integrins, fuse with the plasma membrane, resulting in upregulation of extracellular matrix receptors.

Since rolling leads to intimate contact between neutrophils and endothelial cells, this process should allow neutrophils to become activated by agents expressed on the endothelial cell surface or by substances released from cells lying immediately outside the vasculature. These agents include PAF and oxygen radicals. Interestingly, neutrophils with impaired L-section are able to adhere and emigrate during stasis, possibly bypassing the need for selectin for neutrophil adherence during uterine contractions.

Endothelial cells (EC) can be stimulated[55] by hydrogen peroxide, thrombin and histamine. This leads to the transport of Weibel Palade bodies, which contain P-selectin. Hydrogen peroxide can induce PAF formation on the EC surface. PAF is translocated to the membrane but not released. This transient co-expression of P-selectin and PAF leads to more efficient granulocyte stimulation, and may also lead to cytokine secretion by adhering monocytes. Thus EC stimulation is a fast, receptor-synthesis-independent response occurring within minutes, leading to leukocyte adherence without obligatory PMN activation.

Endothelial cells can be activated[56] by endotoxin and cytokines such as TNF, IL-1 and IL-8, leading to *de novo* expression of E-selectin and adhesion molecules including the endothelial leukocyte adhesion molecule ELAM-1 (with a peak at 4–6 h after stimulation). PAF may also serve as a necessary cofactor, whereas IL-8 may also act as a secondary signalling agent. Shedding of ELAM-1 (soluble ELAM-1) serves as a conventional neutrophil chemoattractant. After binding its neutrophil ligand, ELAM-1 recruits participation of additional adhesion molecules by triggering the activation of integrins on the surface of neutrophils, all of which participate in diapedesis.

As the number of PMNs and macrophages in the near-term cervix increase spectacularly,[2,28] the cascade mechanisms eliciting tissular white blood cell recruitment and activation as described above must be activated before and during delivery. The result of these processes is the benign softening and dilatation of the cervix, as a result of local macrophage and PMN activations permitting the passage of the foetus.

### Cervical ripening

In sheep, cervical division of the cervix from the rest of the uterus still resulted in cervical ripening during labour, suggesting that both processes can occur relatively independently.[57] Cervical $PGE_2$ production increases[58] in response to stretching and it is known from clinical practice that myometrial contractions contribute mechanically to cervical dilatation. Lumbar epidural analgesia transiently reduce uterine contractility, but the rate of cervical dilatation is not affected.[1,59,60] This underscores the relative independence of both processes and the clinically negligible effect of epidural analgesia on the dilatation of the cervix.

The connective tissue of the cervix is composed of collagen, elastin, numerous fibroblasts and relatively few smooth muscle cells, separated by ground substance, which form a strong barrier against foetal loss and ascending bacterial infection.[61] An increase in vascularity, water content and extensive changes in the connective tissue are responsible for clinically recognized cervical softening, effacement and dilatation are encountered as pregnancy progresses. Near term, PMN and macrophages invade cervical connective tissue,[28] leading to a local inflammation described as 'cervicitis'.

*Ground substance:* Proteoglycans form the main component of the ground substance. They are made up of several glycosaminoglycans (GAGs) connected to a protein core. These GAGs contain a large number of sulphate groups and are arranged around the collagen fibrils. Their function is not well understood, but they modify the physical properties of collagen and determine the water content of the cervix. Hyaluronic acid is an important GAG and is associated with the capacity of tissue to retain water. Compared to other GAGs, it binds least strongly with collagen, and thus will act to destabilize the collagen fibrils. Just before labour, the concentration of GAGs increases due to increased synthesis, and during the active phase of labour a decrease in cervical proteoglycans is noted.[62] This drop results in a facilitation of collagen breakdown and an increase in cervical water content. Oxyradicals can elicit the breakdown of ground substance proteins including hyaluronic acid,[63] whereas PMN-derived enzymes for ground substance degradation include cathepsins, elastase and lysozyme. To make it even more complicated, soluble hyaluronic acid can induce the production of some cytokines including TNF[64] and IL-1,[65] and can inhibit oxygen radical formation and phagocytosis[66] by macrophages.

*Collagen:* Cervical collagen, 70% type I and 30% type III,[61] is resistant to most extracellular proteases, except collagenase and PMN or macrophage elastase. During pregnancy, mature collagen, with many cross links is broken down and replaced with new collagen which is more amenable to rapid breakdown at the time of parturition. The amount of intact collagen decreases 70% in comparison with concentrations in the non-pregnant cervix.[67] Local cervical and plasma collagenase enzyme activity is maximal during the active second phase of labour. This collagenase found during labour is synthesized by cervical PMNs, and to a lesser degree, by cervical fibroblasts.[68] It is released as a latent procollagenase, which in turn has to be cleaved by another protease before it is active. Tertiary granules of PMNs contain gelatinase, which hydrolyses denatured collagen and breaks down laminin and fibronectin. This enzyme increases consistently with the influx of PMNs. Interestingly, serum collagenase levels are higher in parturients with ripe cervices who go into preterm labour than in women with unripe cervices who deliver at term.[69]

*Elastin:* The synthesis and degradation of elastin is not well understood,[70] but PMN-derived tissue elastate levels gradually increase during pregnancy, and double again during labour. Moreover, PMN-derived elastase can degrade virtually all extracellular matrix proteins, including collagen and proteoglycans.[27]

In summary, as labour progresses, the increase in proteases outpace the local content of enzyme inhibitor, leading to a net degradation of connective tissue. The major sources of oxygen radicals and proteases are PMNs and macrophages which in their turn are attracted and activated by cytokines. Hormones and prostaglandins alter the sensitivity of these cells to several cytokines. For example, prolactin increases phagocytosis by PMN and macrocytes,[71] possibly leading to accelerated normalization of the cervix postpartum.

### Labour

Identification of hormone receptors, e.g. the oxytocin receptor, and the discovery of regulatory intracellular proteins, helped to clarify the mechanism of the action of relaxing and contracting substances on the myometrium. No studies on the direct effects of cytokines on uterine smooth muscle cells have been published yet. TNF and IL-1 stimulate the production of endothelins, which are potent uterotonics secreted by amnion and endothelial cells, and these cytokines promote the production of several prostaglandins.[17] Endothelins stimulate the production of arachidonic acid by monocytes or macrophages.[72,73]

*The role of nitric oxide:* Nitric oxide (NO)[74] relaxes smooth muscle cells by elevating guanosine 3′5′-cyclic monophosphate (cGMP). It inhibits leucocyte adhesion to endothelial cells[75] and the production of endothelin.[76] NO is synthesized from the L-arginine molecule, a reaction that is catalysed by the enzyme NO-synthase,[74] leaving citrulline as a stable marker of NO synthesis. Nitrite and nitrate are stable end-products of NO metabolism. A first subtype of NO-synthase is a constitutive, cytosolic calcium-dependent enzyme, releasing NO after direct stimulation with calcium ionophores, acetylcholine, bradykinine, lipopolysaccharide, thrombin, PAF and electrical stimulation.[74,77] A second subtype of NO-synthase is an inducible Ca-independent enzyme which is found following cellular contact with cytokines. This upregulation is strongly inhibited by many agents including glucocorticoids, IL-4 and IL-10.[23,78]

In rodents, NO is produced by nerves, blood cells and decidual cells during gestation.[79–81] Nitrate, a stable NO metabolite, plasma cGMP and urinary cGMP are increased in the gravid rat.[82] At the end of gestation and during labour, NO production and the sensitivity of smooth muscle to NO is substantially reduced, which suggests that NO may contribute to the maintenance of uterine quiescence, maternal vasodilatation and uterine immune suppression during gestation but not during labour.[79–81] Administration of an inhibitor of NO-synthase caused hypertension and growth retardation, but, the gestational duration was not affected.[83]

In humans, constitutive NO-synthase is expressed by the syncytiotrophoblast, and inducible NO-synthase can be present on placental chorionic villi and the basal plate of the human uterus.[64] The role of NO during gestation and labour in humans remains to be determined. However, in a small study, transdermal nitrates, which are potent NO sources, seem to inhibit premature labour.[85]

### Hormones, prostaglandins and the immune system

Hormones and prostaglandins are necessary for delivery. Their role during parturition is summarized below, especially with respect to the immune system.

*Oestrogens:* The foetus supplies the placenta with precursors for oestrogen synthesis, and oestrogens are then released into the maternal circulation. Maternal serum levels of oestrogens gradually increase during normal pregnancy. Substances known to augment oestrogen synthesis include intracellular cAMP, human chorionic gonadotropin (hCG), ACTH and insulin, whereas glucocorticoids attenuate oestrogen production.

Oestrogens are thought to be essential in the preparation, rather than the mechanism, of the initiation of birth. In sheep, oestrogens enhance oxytocin responsiveness of the uterus, cause premature parturition, stimulate myometrial activity, increase intracellular calcium and the myometrial content of proteins involved in contraction, increase α- and reduce β-adrenergic receptor content, stimulate prostaglandin production and reverse the inhibitory effects of progesterone. Oestrogens can modulate the synthesis of glycoaminoglycans and can increase the collagen turnover in the uterus. However, conflicting results on the efficacy of oestrogens with respect to cervical ripening have been reported.[86]

*Progesterone:* Progesterone production by the corpus luteum starts early in pregnancy. Thereafter, progesterone is synthesized by the trophoblast. Its production increases with placental size, resulting in a rise in maternal plasma concentration to reach a plateau at about 32 weeks' gestation. Placental progesterone production is thought to operate maximally and at a rate determined by the cholesterol supply from maternal circulation. hCG, β-adrenergic agonists and catecholamines have little effect on progesterone synthesis.[87]

Progesterone can restrict rises in intracellular calcium, restrict the coupling efficiency of β1-adrenergic receptors to enhance cAMP production, or inhibit uterine prostaglandin production. The 'progesterone withdrawal' hypothesis with concomitant oestrogen secretion was based on the dramatic decrease in plasma levels of progesterone just before parturition in several mammalian species. In humans, however, no reduction of plasma progesterone has been observed before the onset of labour. On the other hand, inhibition of progesterone synthesis or surgical progesterone withdrawal result in abortion or increased sensitivity of the myometrium to uterotonic agents.

*Oxytocin:* Oxytocin promotes the production of prostaglandins[88,89] by a protein kinase C dependent mechanism, and oxytocin administration induces labour in near term parturients. However, plasma oxytocin concentrations do not increase initially during labour, and oxytocin does not induce myometrial gap junctions or cervical ripening. Plasma oxytocin levels do not increase until late in parturition, so the increase of membrane oxytocin receptors by an unknown mechanism upregulates the effects of oxytocin during the second stage of labour.[89] Since antoxiban, an oxytocin antagonist, decreased the contraction frequency in patients in preterm labour, oxytocin appears to play a role in the maintenance of contractions in these women.[90]

*The cytokine–neuroendocrine interaction:* The effects of cytokines on the neuroendocrine system are complex.[91] The major site of production of cytokines is the locus of inflammation, viz. the cervix. The cytokines can have their effect either locally, called a paracrine effect, or at a distance, called an endocrine effect. For example, endotoxin or low plasma levels of some cytokines have an effect on the hypothalamus and the pituitary gland.[92] Nevertheless, little information is available about neuroimmune interactions during pregnancy, particularly about the influence of stress and pain during labour on this neuroimmune function.

The hypothalamic–pituitary–adrenal axis is stimulated by IL-1, TNF and to a lesser degree IL-6, mainly via release of corticothrophin-releasing hormone,[93] a direct action on the adrenal gland[94] and probably also on the pituitary gland. This results in an increased production of glucocorticoids.

Severe infection and inflammation are frequently accompanied by an inhibition of reproductive function.[95] Cytokines such as IL-1 and TNF depress the hypothalamic–pituitary–gonadal axis via the hypothalamus (gonadotrophin-releasing hormone), the pituitary (LH, FSH) and the ovary (steroidgenesis).[96] Administration of IL-1 leads to increased oxytocin levels and increases, at least *in vitro*, the uterine myometrial response to oxytocin. However, it should be noted that in contrast to IL-6, neither TNF nor IL-1 are found during normal parturition into the patients' serum.

Glucocorticoids and oestrogens[97] can exert a negative effect on the immune system, because they inhibit the synthesis of IL-1, TNF, IL-2, IL-6 and IL-8 by macrophages. Progesterone is a potent anti-inflammatory agent.[98] Antiprogestins ameliorate cervical ripening, as these agents promote PMN influx,[99] probably as a result of a neutralization of an inhibitory effect of progesterone on IL-8 production by choriodecidual cells.[14]

*Prostaglandins:* Some prostaglandins given by mouth, intravenously or by cervical instillation, evoke myometrial contractions, cervical ripening and abortion or delivery at any stage of pregnancy. Inhibitors of prostaglandin synthesis lengthen the induction–abortion time interval after instillation of hypertonic saline, or lead to prolongation of gestation.

Arachidonic acid in tissue is present in an esterified form in glycerophospholipids, which form 10 to 30% of the total fatty acid content of the (intra)uterine tissues. The formation of free arachidonic acid is considered to be a rate-limiting stop in prostaglandin synthesis. In some tissues, however, the rate of prostaglandin production is at least partially regulated by the rate of conversion of arachidonic acid to prostaglandins.[86] Moreover, not all arachidonic acid is necessarily directed toward prostaglandin and prostanoid biosynthesis, because a significant proportion of it may be converted by lipoxygenase to form leukotrienes and hydroxyeicosatetraenoic acids, by epoxygenase to form epoxides and, more importantly, to reincorporation in the structural fat of cell membranes.

During the production of prostaglandins from arachidonic acid, oxygen radicals are formed. These radicals can influence the cyclooxygenase enzyme, altering PG formation.[29,30]

Two genes, for cyclooxygenase, termed cox-1 and cox-2, have been cloned and expressed in functional form.[100] The cox-1 gene is expressed ubiquitously *in vivo* and *in vitro*, whereas the cox-2 gene is expressed at very low levels in normal tissues. The expression of the cox-2 gene is induced by lipopolysaccharide or IL-1. The cyclooxygenase undergoes irreversible self-inactivation, so that modulation of activity depends on continued synthesis of the enzyme.[101] Intrauterine bacterial inoculation in mice led to preterm delivery, accompanied by induction of ribonucleic acid transcript for several cytokines and for cox-2.[18] Cox-2 knockout mice are infertile, which may at least in part be related to the important role which prostaglandins play in implantation, pregnancy and lactation.[102]

Bacterial toxins, particularly IL-1, TNF and IL-6, in concentrations found in amniotic fluid during parturition,[10] increase prostaglandin formation by amnion cells and chorion laeve cells ($PGE_2$, $PGF_{2\alpha}$),[12] endometrial stromal cells which are the progenitors of the decidual cells ($PGF_{2\alpha}$, $PGE_2$)[103,104] and myometrial muscle cells ($PGI_2$, $PGE_2$ and $PGF_{2\alpha}$).[9] Other cytokines, such as IL-4[105] and transforming growth factor-β2[43,106] suppress prostaglandin production by monocytes. Therefore, infection as a causative event of labour has led to the theory that part of the problem of prematurity is a problem of infection.[10,11,107] Some studies indicate a modulating effect of prostaglandins on immune cells with respect to cytokine production, or response threshold to some cytokines.[14] Recently, uteroglobulin (Clara Cell protein, CC10, protein 1) is found in abundance in amniotic fluid[108] and probably originates from the foetal lung. It has potent anti-inflammatory properties by inhibiting phospholipase $A_2$ and PAF generation;[109] however, its exact role in parturition is under investigation.

### Conclusion

The physiology of parturition is at least partially explained by the contribution of the immune system. Several cascades seem to play a role, but no pathway is totally independent. Before therapeutic intervention aimed at modulating one or more cascades to reduce cervical ripening and thus to prevent prematurity can be considered, one must be certain of the side effects of such therapy. For example, antibodies against ELAM will dramatically reduce PMN invasion of the cervix, inhibiting cervical softening of dilatation. However, the resistance of the mother against bacterial invasion will be reduced simultaneously, leading to bacteraemia with potentially disastrous effects on both the mother and foetus.

Extensive research is necessary to understand the pathophysiology of parturition to allow appropriate interference with these physiological pathways. Moreover, the pathophysiological backgrounds of several gestationally related diseases, including haemolysis, elevated liver enzymes, low platelet or HELLP syndrome, (pre)eclampsia or recurrent abortion, need to be unravelled. Further research to determine the effects of analgesia and anaesthesia on this immuno-obstetric system will also identify more accurately the hazards or benefits of these techniques on foetal and maternal well-being.

### References

1. Finster M. Effects of epidural analgesia on the progress of labor and the mode of delivery. *Eur J Obstet Gynecol Reprod Biol* 1995; **59**: S31–S33.
2. Liggings GC. Ripening of the cervix. *Semin Perinatol* 1978; **2**: 261–271.
3. Laham N, Rice GE, Bishop GJ, Ransome C, Brennecke SP. Interleukin 8 concentrations in amniotic fluid and peripheral venous plasma during human pregnancy and parturition. *Acta Endocrinol* 1993; **129**: 220–224.
4. Romero R, Ceska M, Avila C, Mazor M, Behnke E, Lindley I. Neutrophil attractant/activating peptide-1/inerleukin-8 in term and preterm parturition. *Am J Obstet Gynecol* 1991: **165**: 813–820.
5. Saito S, Kasahara T, Kato Y, Ishihara Y, Ichijo M. Elevation of amniotic fluid interleukin 6 (IL-6), IL-8 and granulocyte colony stimulating factor (G-CSF) in term and preterm parturition. *Cytokine* 1993; **5**: 81–88.
6. Cherouny PH, Pankuch GA, Romero R, *et al.* Neutrophil attractant/activating peptide-1/interleukin-8: association with histologic chorioamnionitis, preterm delivery, and bioactive amniotic fluid leukoattractants. *Am J Obstet Gynecol* 1993; **169**: 1299–1303.
7. De Jongh R, Puylaert M, Ombelet W, Heylen R, Bosmans E. The effect of anaesthetic techniques in peripartal maternal interleukin-6 levels. *Br J Anaesth* 1994; **72**: S100.
8. Opsjon SL, Wathen NC, Tingulstad S, *et al.* Tumor necrosis factor, interleukin-1, and interleukin-6 in normal human pregnancy. *Am J Obstet Gynecol* 1993; **169**: 397–404.
9. Casey ML, Cox SM, Word A, MacDonald PC. Cytokines and infection-induced preterm labour. *Reprod Fertil Dev* 1990; **2**: 499–510.
10. Romero R, Brody DT, Oyarzum E, *et al.* Infection and labor. III Interleukin-1: a signal for the onset of parturition. *Am J Obstet Gynecol* 1989; **160**: 1117–1123.
11. Bennett PR, Rose MP, Myatt L, Eleber MG. Preterm labor: stimulation of arachidonin and metabolism in human amnion by bacterial products. *Am J Obstet Gynecol* 1987; **156**: 649–655.
12. Lundin-Schiller S, Mitchell MD. Prostaglandin production by human chorion laeve cells in response to inflammatory mediators. *Placenta* 1991; **12**: 353–363.
13. Barclay CG, Brennand JE, Kelly RW, Calder AA. Interleukin-8 production by the human cervix. *Am J Obstet Gynecol* 1993; **169**: 625–632.
14. Kelly RW, Leask R, Calder AA. Choriodecidual production of interleukin-8 and mechanism of parturition. *Lancet* 1992; **339**: 776–777.
15. Dudley DJ, Trautman MS, Mitchell MD. Inflammatory mediators regulate interleukin-8 production by cultured gestational tissues: evidence for a cytokine network at the choriodecidual interface. *J Clin Endocrinol Metab* 1993; **76**: 404–410.
16. Trautman MS, Dudley DJ, Edwin SS, Collmer D, Mitchell MD. Amnion cell biosynthesis of interleukin-8: regulation by inflammatory cytokines. *J Cell Physiol* 1992; **153**: 38–43.
17. Mitchell MD, Trautman MS, Dudley DJ. Immunoendocrinology of preterm labour and delivery. *Baillière's Clin Obstet and Gynecol* 1993; **7**: 533–575.
18. Hirsch E, Saotome I, Hirsh D. A model of intrauterine infection and preterm delivery in mice. *Am J Obstet Gynecol* 1995; **172**: 1598–1603.
19. Roitt I. The basis of immunology. In: *Essential Immunology. 7th Edition.* Oxford: Blackwell Scientific Publications, 1991.
20. Van Furth R, Cohn ZA, Hirsch JG, Humphrey JH, Spector WG, Langevoort HL. The mononuclear phagocyte system. A new classification of macrophages, monocytes and their precursor cells. *Bull WHO* 1972; **46**: 845–852.
21. Kunkel SL, Standiford T, Kasahara K, Strieter RM. Interleukin-8 (IL-8): the major neutrophil chemotactic factor in the lung. *Exp Lung Res* 1991; **17**: 17–23.
22. Sibille Y, Merrill WW, Naegel GP, Care SB, Cooper JA, Reynolds HY. Human alveolar cells release *in vitro* a factor(s) which inhibits neutrophil chemotaxis. *Am J Cell Mol Biol* 1989; **1**: 407–416.
23. Jorens PG, Matthys KE, Bult H. Modulation of nitric oxide synthase activity in macrophages. *Mediators of Inflammation* 1995; **4**: 75–89.
24. Bulmer JN, Morrison L, Smith JC. Expression of class II MHC gene products by macrophages in human uteroplacental tissue. *Immunology* 1988; **63**: 707–714.
25. Vince GS, Starkey PM, Jackson MC, Sargent IL, Redman CW. Flow cytometric characterisation of cell populations in human pregnancy decidua and isolation of decidual macrophages. *J Immunological Methods* 1990; **132**: 181–189.
26. Starkey PM. The decidua and factors controlling placentation. In: Redman CW, Sargent IL, Starkey PM, eds. *The Human Placenta.* Oxford, UK: Blackwell Scientific Publications, 1993; 362–413.
27. Johnson KJ, Varani J, Smolen JE. Neutrophil activation and function in health and disease. In: Coffey RG, ed. *Granulocyte Responses to Cytokines.* New York: Marcel Dekker, Inc., 1992; 1–46.
28. Epperson JW, Hellman LM, Galcin GA, Busby T. The morphological changes in the cervix during pregnancy, including intra-epithelial carcinoma. *Am J Obstet Gynecol* 1951; **61**: 50.
29. Schoenberg MH, Paes E. Intestinal ischemia and reperfusion. *Progress in Applied Microcirculation* 1989; **13**: 109–125.
30. Sanderud J, Bjoro K, Saugstad OD. Oxygen radicals stimulate thromboxane and prostacyclin synthesis and induce vasoconstriction in pig lungs. *Scand J Clin Lab Invest* 1993; **53**: 447–455.
31. Mela-Riker L, Tavakoli H. Mitochondrial function in shock. *Am J Emerg Med* 1983; **2**: 2–7.
32. McCord JM. Oxygen-derived free radicals in postischemic tissue injury. *N Engl J Med* 1985; **312**: 159–163.
33. Morel F, Doussière J, Vignais PV. The superoxide-generating oxidase of phagocytic cells. Physiological, molecular, and pathological aspects. *Eur J Biochem* 1991; **201**: 523–546.
34. Babior BM. Oxidants from phagocytes: agents of defense and destruction. *Blood* 1984; **64**: 959–966.
35. Gardner HW. Oxygen radical chemistry of polyunsaturated fatty acids. *Free Rad Biol Med* 1989; **7**: 65–86.
36. Imlay JA, Linn S. DNA damage and oxygen radical toxicity. *Science* 1988; **240**: 1302–1309.
37. Davies KJA. Protein damage and degradation by oxygen radicals. *J Biol Chem* 1987; **262**: 8895–9901.
38. Kauma SW, Matt D, Strom S, Eierman D, Turner T. Interleukin-1β, human leukocyte antigen, HLA-DRα and transforming growth factor β expression in endometrium, placenta and placental membranes. *Am J Obstet Gynecol* 1990; **153**: 1430–1437.
39. Berkowitz RS, Faris HM, Hill JA, Anderson DJ. Localization of leucocytes and cytokines in chorionic villi of normal placentas and complete hydatiform males. *Gynecol Oncol* 1990; **37**: 396–400.
40. Scales WE. Structure and function of interleukin-1. In: Kinkel SL, Remick DG, eds. *Cytokines in Health and Disease.* New York: Marcel Dekker, 1992; 15–26.
41. Ito A, Hiro D, Ojima Y, Mosi Y. Spontaneous production of interleukin-1-like factors from pregnant rabbit uterine cervix. *Am J Obstet Gynecol* 1988; **159**: 261–265.
42. Romero R, Mazor M, Tartakovsky B. Systemic administration of interleukin-1 induces preterm parturition in mice. *Am J Obstet Gynecol* 1991; **165**: 969–971.
43. Bry K, Hallman M. Transforming growth factor-beta 2 prevents preterm delivery induce body interleukin-1 alpha and tumor necrosis factor-alpha in the rabbit. *Am J Obstet Gynecol* 1993; **168**: 1318–1322.
44. Romero R, Tartakovsky B. The natural interleukin-1 receptor antagonist prevents interleukin-1-induced preterm delivery in mice. *Am J Obstet Gynecol* 1992; **167**: 1041–1045.
45. Cox G, Gauldie J. Structure and function of interleukin-6. In: Kunkel SL, Remick DG, eds. *Cytokines in Health and Disease.* New York: Marcel Dekker, Inc., 1992; 97–120.
46. Camann WR, Hortvet LA, Hughes N, Bader AM, Datta S. Maternal temperature regulation during extradural analgesia for labour. *Br J Anaesth* 1991; **67**: 565–568.
47. Puylaert MJ, De Jongh RF, Ombelet W, *et al.* The effect of epidural analgesia during labor on the immune system: neonatal blood interleukin-6 levels. *Int J Obstet Anesth* 1994; **3**: 116–117.
48. Laham N, Rice G, Bishop G, Hansen M, Bendizen K, Bennecke G. Elevated plasma interleukin-6: a biochemical marker of human preterm labour. *Gynecol Obstet Invest* 1993; **36**: 145–147.
49. Tsuji Y, Torti F. Tumour necrosis factor: structure and function. In: Kunkel SL, Remick DG, eds. *Cytokines in Health and Disease.* New York: Marcel Dekker, 1992; 131–150.
50. Van Damme J, Interleukin-8 and related molecules. In: Thompson AW, ed. *Cytokine Handbook.* London: Academic Press, 201–214.
51. Jorens PG, Richman-Eisenstat JB, Housset BP, *et al.* Interleukin-8 induces neutrophil accumulation but not protease secretion in the canine trachea. *Am J Physiol* 1992; **263**: L708–L713.
52. Colditz IG. Effect of endogenous prostaglandin E2 and actinomyocin D on plasma leakage induced by neutrophil activating peptide-1/interleukin-8. *Immunol Cell Biol* 1990; **68**: 397–403.
53. Granger DN, Kubes P. The microcirculation and inflammation: modulation of leucocyte-endothelial cell adhesion. *J Leukocyte Biol* 1994; **55**: 662–675.
54. Wardlaw A. Leucocyte adhesion to endothelium. *Clin Exp Allergy* 1990; **20**: 619–626.
55. Lewis MS, Whatley RE, Cain P, McIntyre TM, Prescott SM, Zimmerman GA. Hydrogen peroxide stimulates the synthesis of platelet activating factor by endothelium and induces endothelial cell dependent neutrophil adhesion. *J Clin Invest* 1988; **82**: 2045–2055.
56. Pober JS, Cotran RS. The role of endothelial cells in inflammation. *Transplantation* 1990; **50**: 537–544.
57. Ledger WL, Webster M, Harrison LP. Increase in cervical extensibility during labour induced after isolation of the cervix from the uterus in pregnant ewes. *Am J Obstet Gynecol* 1985; **151**: 397–402.
58. Hillier K, Coad N. Synthesis of prostaglandins by the human uterine cervix *in vitro* during passive mechanical stretch. *J Pharmacol* 1982; **34**: 262–263.
59. Craft JB, Epstein BS, Coakley CS. Effect of lidocaine with epinephrine versus lidocaine (plain) on induced labor. *Anesth Analg* 1972; **51**: 243–246.
60. Vasicka A, Kretchmer H. Effect of conduction and inhalation anesthesia on uterine contractions. *Am J Obstet Gynecol* 1961; **82**: 609–611.
61. Danforth DN. The morphology of the human cervix. *Clin Obstet Gynecol* 1983; **26**: 7–13.
62. Osmers R, Rath W, Pflanz MA, Kuhn W, Stuhlsatz HW, Szeverényl M. Glycosaminoglycans in cervical tissue during pregnancy and parturition. *Obstet Gynecol* 1993; **81**: 88–92.
63. Bast A, Goris RJA, Oxidative stress. Biochemistry and human disease. *Pharm Weekbl [Sci]* 1989; **11**: 199–206.
64. Noble PW, Lake FR, Henson PM, Riches DW. Hyaluronate activation of CD44 induces insulin-like growth factor-1 expression by a tumor necrosis-alpha-dependent mechanism in murine macrophages. *J Clin Invest* 1993; **91**: 2368–2377.
65. Hiro D, Ito A, Matsuta K, Mori Y. Hyaluronic acid is an endogenous inducer of interleukin-1 production by human monocytes and rabbit macrophages. *Biochem Biophys Res Commun* 1986; **140**: 715–722.
66. Suzuki Y, Yamaguchi T. Effects of hyaluronic acid on macrophage phagocytosis and active oxygen release. *Agents Actions* 1993; **38**: 32–37.
67. Ekman G, Almstrom H, Granstrom L, Malmstrom A, Norman M, Woessner JF. Connective tissue in human cervical ripening. In: Leppert PC, Woessner JF, eds. *The Extracellular Matrix of Uterus, Cervix and Fetal Membranes.* Ithaca, NY: Perinatology Press, 87–96.
68. Osmers R, Rath W, Adelmann-Grill BC, *et al.* Origin of cervical collagenase during parturition. *Am J Obstet Gynecol* 1992; **166**: 1455–1460.
69. Rajabi MR, Dean DD, Woessner JFJ. High levels of serum collagenase in premature labor—a potential biochemical marker. *Obstet Gynecol* 1987; **69**: 179–186.
70. Gunja Smith Z, Woessner JF. Content of collagen and elastin cross-links pyridinoline and the desmosines in the human uterus in various reproductive states. *Am J Obstet Gynecol* 1985; **153**: 92–95.
71. Thorat SP, Thatte UM, Pai N, Dahanuker SA. Effect of prolactin on neutrophils and monocytes. *Acta Haematol* 1993; **89**: 219–220.
72. Helset E, Sildnes T; Selijelid R, Konopski Z. Endothelin-1 stimulates human monocytes *in vitro* to release TNF-α, IL-1β and IL-6. *Mediators of Inflammation* 1993; **2**: 417–422.

73. Millud V, Lagente V, Gillardeoux O, *et al.* Activation of guinea pig alveolar macrophages by endothelin-1. *J Cardiovasc Pharm* 1991; **17(Suppl 7)**: S233–S235.
74. Moncada S, Palmer RMG, Higgs EA. Nitric oxide: physiology, pathophysiology and pharmacology. *Pharmacol Rev* 1991; **443**: 109–142.
75. Kubes P, Suzuki M, Granger DN. Nitric oxide: an endogenous modulator of leukocyte adhesion. *Proc Natl Acad Sci* 1991; **88**: 4651–4655.
76. Boulanger C, Luscher TF. Release of endothelin from the porcine aorta. Inhibition by endothelium-derived nitric oxide. *J Clin Invest* 1990; **85**: 587–590.
77. Nathan C. Nitric oxide as a secretory product of mammalian cells. *FASEB J* 1992; **6**: 3051–3064.
78. Oswald IP, Gazzinelli RT, Sher A, James SL. IL-10 synergizes with IL-4 and transforming growth factor-beta to inhibit macrophage cytotoxic activity. *J Immunol* 1992; **148**: 3578–3582.
79. Natuzzi ES, Ursell PC, Harrison M, Buscher C, Riemer RK. Nitric oxide synthase activity in the pregnant uterus decreases at parturition. *Biochem Biophys Res Commun* 1993; **194**: 1–8.
80. Sladek SM, Regenstein AC, Lykins D, Roberts JM. Nitric oxide synthase activity in pregnant rabbit uterus decreases on the last day of pregnancy. *Am J Obstet Gynecol* 1993; **169**: 1285–1291.
81. Yallampalli C, Garfield RE, Byam-Smith M. Nitric oxide inhibits uterine contractility during pregnancy but not during delivery. *Endocrinology* 1993; **133**: 1899–1902.
82. Conrad KP, Joffe GM, Kruszyna H, *et al.* Identification of increased nitric oxide biosynthesis during pregnancy in rats. *FASEB J* 1993; **7**: 566–571.
83. Yallampalli C, Garfield RE. Inhibition of nitric oxide synthesis in rats during pregnancy produces signs similar to those of preeclampsia. *Am J Obstet Gynecol* 1993; **169**: 1316–1320.
84. Morris NH, Eaton BM, Sooranna SR, Steer PJ. NO synthase activity in placental bed and tissues from normotensive pregnant women. *Lancet* 1993; **342**: 679–680.
85. Lees C, Campbell S, Jauniaux E, *et al.* Arrest of preterm labour and prolongation of gestation with glyceryl trinitrate, a nitric oxide donor. *Lancet* 1994; **343**: 1325–1326.
86. Bernal AL, Watson SP, Phaneuf S, Europe-Finner GN. Biochemistry and physiology of preterm labour and delivery. *Baillière's Clin Obstet Gynecology* 1993; **7**: 523–552.
87. Casey ML, Macdonald PC. Placental endocrinology. In: Redman CW, Sargent IL, Starkey PM, eds. *The Human Placenta.* Oxford, UK: Blackwell Scientific Publications, 1993.
88. Fuchs AR, Husslein P, Fuchs F. Oxytocin and the initiation of human parturition II. Stimulation of prostaglandin production in human decidua by oxytocin. *Am J Obstet Gynecol* 1981; **141**: 694–699.
89. Fuchs AR, Fuchs F, Hussein P, Soloff MA, Fernström MJ. Oxytocin receptors and human parturition: a dual role for oxytocin in the initiation of labor. *Science* 1982; **1982**: 1396–1398.
90. Goodwin TM, Paul R, Silver H, *et al.* The effect of the oxytocin antagonist antoxiban on preterm activity in the human. *Am J Obstet Gynecol* 1994; **170**: 474–478.
91. Imura H, Fukata J, Mori T. Cytokines and endocrine function: an interaction between the immune and neuroendocrine systems. *Clin Endocrinology* 1991; **35**: 107–115.
92. Naito Y, Fukata J, Shindo K, *et al.* Effect of interleukins on plasma arginine, vasopressin and oxytoxin levels in conscious, freely moving rats. *Biochem Biophys Res Comm* 1991; **174**: 1189–1195.
93. Sapolsky R, Rivier C, Yamamoto G, Plotsky P, Vale W. Interleukin-1 stimulates the secretion of hypothalamic corticotropin releasing factor. *Science* 1987; **238**: 522–524.
94. Taminaga T, Fukata J, Naito Y, *et al.* Prostaglandin-dependent *in vitro* stimulation of adrenocortical steroidgenesis by interleukins. *Endocrinology* 1991; **128**: 526–531.
95. Rivier C, Vale W. In the rat, interleukin-α acts at the level of brain and the gonads to interfere with gonadotropin and sex steroid secretion. *Endocrinology* 1989; **124**: 2105–2109.
96. Fukuoka M, Yanuda K, Fujiwara H, Kanzaki H, Mori T. Interaction between interferon gamma, tumour necrosis factor alpha and interleukin-1 in modulating progesterone and oestradiol production by human luteinized granula cells in culture. *Hum Reprod* 1992; **7**: 1361–1364.
97. Li ZG, Danis VA, Brooks PM. Effects of gonadal steroids on the production of IL-1 and IL-6 by blood mononuclear cells *in vitro*. *Clin Exp Rheumatol* 1993; **11**: 157–162.
98. Siiteri SJ, Febres F, Clemens LE, Chang RJ, Gondos D, Stites D. Progesterone and maintenance of pregnancy: is progesterone nature's immunosuppressant? *Ann NY Acad Sci* 1977; **286**: 384–397.
99. Chwalisz K. Cervical ripening and induction of labour with progesterone antagonists. 1988.
100. Hia T, Ristimaki A, Appleby S, Barriocanal JG. Cylo-oxygenase gene expression in inflammation and angiogenesis. *Ann NY Acad Sci* 1993; **696**: 147–204.
101. Smith WL. Prostanoid biosynthesis and mechanism of action. *Am J Physiol* 1992; **263**: F181–F191.
102. Dinchuk JE, Car BD, Focht RJ, *et al.* Renal abnormalities and an altered inflammatory response in mice lacking cyclooxygenase II. *Nature* 1995; **378**: 406–409.
103. Jacobs AL, Carson DD. Uterine epithelial cell secretion of interleukin-1 alpha induces prostaglandin E2 (PGE2) and PGF2 alpha secretion by uterine stromal cells *in vitro*. *Endocrinology* 1993; **132**: 300–308.
104. Mitchell MD, Edwin SS, Romero RJ. Prostaglandin biosynthesis by human decidual cells: effects of inflammatory mediators. *Prostaglandins Leukot Essent Fatty Acids* 1990; **41**: 35–38.
105. Corcoran ML, Stetler-Stevensen WG, Brown PD, Wahl LM. Interleukin-4 inhibition of prostaglandin E2-synthesis blocks interstitial collagenase and 92-kDa type IV collagenase/gelatinase production by human monocytes. *J Bio Clin* 1992; **261**: 515–519.
106. Bry K, Hallman M. Transforming growth factor-beta opposes the stimulatory effects of interleukin-1 and tumor necrosis factor on amnion cell prostaglandin E2 production: implications for preterm labor. *Am J Obstet Gynecol* 1992; **167**: 222–226.
107. Kirschbaum T. Antibiotics in the treatment of preterm labor. *Am J Obstet Gynecol* 1993; **168**: 1239–1246.
108. Bernard A, Thielemans N, Lauwerys R, Langhendries JP, Van Lierde M, Freund M. Clara cell protein in human amniotic fluid: a potential marker of fetal lung growth. *Ped Res* 1994; **36**: 771–775.
109. Miele L, Cordella-Miele E, Mantile G, Peri A, Mukherjee AB. Uteroglobin and uteroglobin-like proteins: the uteroglobin family of proteins. *J Endocrinol Invest* 1994; **17**: 679–692.

**Received 10 January 1996; accepted in revised form 9 April 1996**
